# Primary cavernous hemangioma of the thyroid mimicking an ectopic cervical thymoma

**DOI:** 10.1093/jscr/rjac411

**Published:** 2022-09-30

**Authors:** Alberto Díaz-García, Eugenia Caballero-Rodríguez, Rubén García-Martínez, Julio Jordán Balanzá, Manuel Ángel Barrera Gómez

**Affiliations:** General Surgery Unit, Hospital Universitario Nuestra Señora de Candelaria, Tenerife, Spain; Head and Neck Surgery Unit, Hospital Universitario Nuestra Señora de Candelaria, Tenerife, Spain; Head and Neck Surgery Unit, Hospital Universitario Nuestra Señora de Candelaria, Tenerife, Spain; Head and Neck Surgery Unit, Hospital Universitario Nuestra Señora de Candelaria, Tenerife, Spain; General Surgery Unit, Hospital Universitario Nuestra Señora de Candelaria, Tenerife, Spain

**Keywords:** thyroids, hemangioma, neck surgery

## Abstract

Hemangiomas are one of the most common soft tissue tumors, most of which are located in the skin and subcutaneous tissue. However, a primary thyroid hemangioma is extremely infrequent, so there are only a few cases described in the current literature. As the clinical presentation and characteristics in the imaging tests are non-specific, it is difficult to obtain a preoperative diagnosis. In most cases, the definitive diagnosis is achieved by the histological examination. Due to the rarity of this tumor, we described the case of a 51-year-old female patient affected by thyroid cavernous hemangioma, the differential diagnosis that we considered and a review of the current literature.

## INTRODUCTION

Hemangiomas are tumors of vascular origin, with their main characteristic being their important capillary proliferation. Most of them have their origin in the skin, the oral cavity and the liver [1]. Primary thyroid hemangiomas are rare, with <40 cases described in the literature [2], and the first being described by Pickleman *et al*. [3] in 1975. Because the clinic is nonspecific and there are not any suggestive imaging features in ultrasound (US) or computed tomography (CT) [4], preoperative diagnosis is difficult.

## CASE REPORT

**Figure 1:**
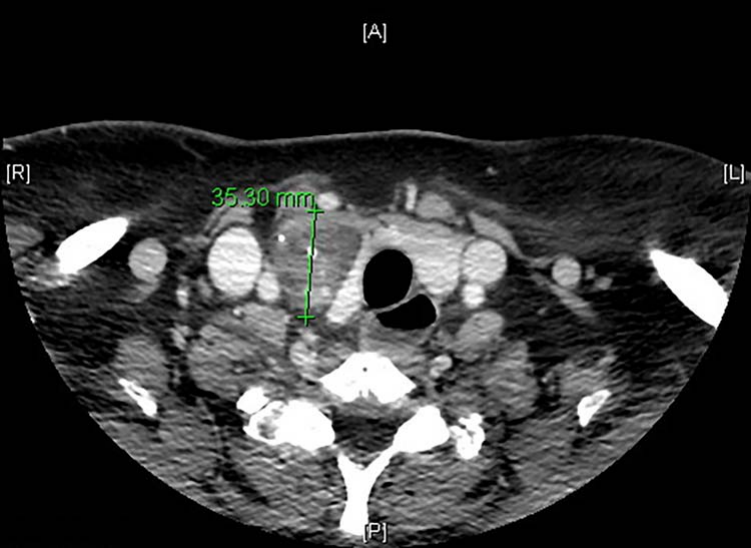
Axial CT scan image showing a right thyroid nodular lesion, discovered incidentally.

We present the case of a 51-year-old female patient who was recently diagnosed with myasthenia gravis and consults with a right thyroid nodular lesion, discovered incidentally in a CT. The initial suspicion by imaging is ectopic thymoma. It is a well-defined, hypodense nodule, 4.5 × 3.3 × 2.5 cm, associated with intralesional calcifications, which seems to compress the right thyroid lobe extrinsically (Fig. 1). However, more cranially, it seems to present a union pole with the right lobe, which could correspond to an eccentric thyroid nodule. We carried out adirected study of the neck and thyroid. The patient denied compressive symptoms, dyspnea or voice changes. On examination, the patient presented a palpable nodule in the right thyroid lobe, soft and mobile. US study shows a hypoechoic heterogeneous nodular image, 5.5 × 3.1 cm in diameter, with initial diagnostic doubts of its thyroid origin (categorized as TIRADS 3), and probably related to a possible thymoma. A fine needle aspiration (FNA) is performed, with an insufficient sample result (Bethesda 1) and a smear of hematic material without representative epithelial cellularity. The case is assessed by a multidisciplinary committee, surgical treatment is recommended, with a diagnostic/therapeutic aim. In the intervention, a hypervascularized lesion dependent on the right thyroid lobe is evidenced and a right hemithyroidectomy and isthmectomy are carried out, with preservation of the parathyroid glands and neuromonitoring of the recurrent laryngeal nerve. She presents a favorable post-operative period, without any incident.

**Figure 2:**
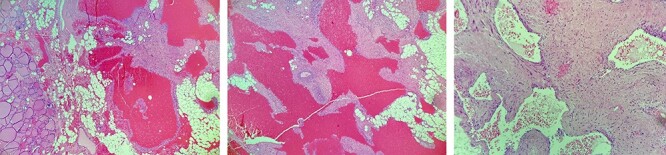
Histological images, showing ectatic vascular spaces filled with blood, lined by flat endothelial cells without atypia and also areas with variable fibrosis, all compatible with a cavernous hemangioma of thyroid origin.

Pathological anatomy describes a well-circumscribed nodular lesion, consisting of ectatic vascular spaces filled with blood and lined by flat endothelial cells without atypia (Fig. 2). In addition, areas with variable fibrosis are identified, without histological signs of malignancy, all compatible with a cavernous hemangioma of thyroid origin. As an incidental finding, a well-differentiated 1.5-mm papillary microcarcinoma with free margins and no vascular or perineural invasion is found. With these results, the case is discussed again in a multidisciplinary committee, which decided follow-up. Finally, the treatment of the original pathology, myasthenia gravis, is completed, with videolaparoscopic thymectomy. The post-operative period is uneventful and the pathological anatomy showed no remarkable findings.

## DISCUSSION

Hemangioma is a benign tumor caused by vascular endothelial cell proliferation or vascular malformation and is classified histologically as synovial, cavernous, capillary, venous, racemose or arteriovenous [4]. It is one of the most common soft tissue tumors, most of which are located in the skin and subcutaneous tissue [5]. However, it can affect other organs and tissues such as liver, gastrointestinal tract, muscle tissue or bone tissue [5]; its presence in thyroid tissue is extremely infrequent. It is considered that these lesions associate their origin with an abnormal development conditioned by a functional incapacity of the angioblastic mesenchyme [5]. There is an association between the presence of hemangiomas in different locations and some hereditary syndromes such as Rendu-Weber-Osler, Parke-Weber-Klippel, Hippel-Lindau, Sturge–Weber or Maffuci syndrome [6].

Primary hemangiomas in the thyroid gland are infrequent, given the scarcity of cases described in the literature, since Pickleman *et al*. [3] presented the first case of thyroid hemangioma in 1975. They can also have their origin in traumatic events at the cervical level or in biopsies through fine needle puncture, which condition abnormal vascular proliferation after the formation of organized hematomas [7]. According to the cases described, there is evidence of a slight prevalence in men, with a highly variable age between 4 and 80 years, as with regard to the size of the lesion, with a range between 2 and 22 cm [4].

The clinical presentation of these lesions is nonspecific, often appearing simply as the growth of a cervical mass. In asymptomatic patients, these are detected as incidental findings in imaging tests requested as part of the study of other pathologies, as in is our case. Since it is a vascularized lesion, there is a risk of intralesional bleeding and consequently an increase in volume which can condition compressive clinical manifestations [8].

Obtaining a preoperative diagnosis is very difficult due to the absence of specific characteristics in the imaging tests and in the histological study by FNA [4]. In most cases, for the differential diagnosis of thyroid disease, the initial diagnostic test is US. In general, they are described as heterogeneous and hypoechoic lesions, well defined and with different morphologies [9]. In addition, this type of tumor can present multiple linear septa due to the presence of intralesional vascular channels [10]. The progressive dilation of these vascular channels associated with slow blood flow predisposes to the appearance of thrombosis and phleboliths [11], and calcifications may also appear that may question the benignity of the lesion. Sometimes other diagnostic tests are used, such as magnetic resonance imaging, digital subtraction angiography, single photon emission CT or red blood cell scintigraphy in which the recorded signal intensity and the different intralesional vascular patterns can sometimes support the preoperative diagnosis [5, 12]. Cavernous hemangiomas are characterized by the absence or slight uptake of contrast due to poor perfusion secondary to the slow flow and consequent late filling [13]. The profitability of FNA is very limited, since in most cases, blood is obtained rather than the epidermal cell components [4].

Surgery appears mandatory for a definitive and confirmatory diagnosis, given the need of a histological study. Hemithyroidectomy or total thyroidectomy will be assessed based on preoperative suspicion of malignancy, compressive symptoms or retrosternal extension [14]. Given the characteristics of the tumor, there is a risk of intraoperative bleeding directly related to the size of the tumor, so an adequate preoperative evaluation is required [3].

Hemangiomas are tumors made up of soft tissue, circumscribed, with ectatic vascular spaces filled with blood delimited by flat or cubic endothelial cells, with the occasional presence of atypia and papillary endothelial projections [10, 15]. Within the differential diagnosis of this type of lesions, there is the infrequent thyroid lymphangioma, papillary endothelial hyperplasia and angiosarcoma [16].

In our case, given the history of myasthenia gravis, cervical ectopic thymoma was initially considered as a possible differential diagnosis. Cervical ectopic thymomas are extremely rare as well, with an unknown incidence and <30 cases described in the literature [17, 18]. Most are asymptomatic, presenting as a nonspecific cervical mass which often raises the differential diagnosis with thyroid-dependent masses. In 30–50% of cases, it can be associated with parathymic syndromes, the most frequent of which is myasthenia gravis [19].
